# 1-[3,5-Bis(4-chloro­phen­yl)-4,5-dihydro-1*H*-pyrazol-1-yl]ethanone

**DOI:** 10.1107/S1600536810025584

**Published:** 2010-07-07

**Authors:** Jerry P. Jasinski, Albert E. Pek, S. Samshuddin, B. Narayana, H. S. Yathirajan

**Affiliations:** a Department of Chemistry, Keene State College, 229 Main Street, Keene, NH 03435-2001, USA; b Department of Studies in Chemistry, Mangalore University, Mangalagangotri 574 199, India; c Department of Studies in Chemistry, University of Mysore, Manasagangotri, Mysore 570 006, India

## Abstract

In the title compound, C_17_H_14_Cl_2_N_2_O, the dihedral angles between the pyrazole ring and the mean planes of the benzene and chloro-substituted benzene rings are 75.97 (1) and 16.63 (1)° respectively. In the crystal, two weak C—H⋯O inter­molecular hydrogen bonds and π–π stacking inter­actions [centroid–centroid distances = 3.774 (4) and 3.716 (7) Å] are observed.

## Related literature

For the anti­tumor, anti­bacterial, anti­fungal, anti­viral, anti­parasitic, anti-tubercular and insecticidal properties of substituted pyrazolines, see: Hes *et al.* (1978 ▶); Manna *et al.* (2005 ▶); Amir *et al.* (2008 ▶). For their anti-inflammatory, anti-diabetic, anaesthetic and analgesic properties, see: Regaila *et al.* (1979 ▶). For their use in organic synthesis, see: Klimova *et al.* (1999 ▶); Bhaskarreddy *et al.* (1997 ▶). For a continuation of the work on pyrazoline derivatives, see: Samshuddin *et al.* (2010 ▶); Fun *et al.* (2010 ▶); Yathirajan *et al.* (2007*a* ▶,*b* ▶); Butcher *et al.* (2007 ▶). For related structures, see: Jian & Wang (2006 ▶); Anuradha *et al.* (2008 ▶); Lu *et al.* (2008 ▶); Jian *et al.* (2006 ▶); Wang *et al.* (2005 ▶).
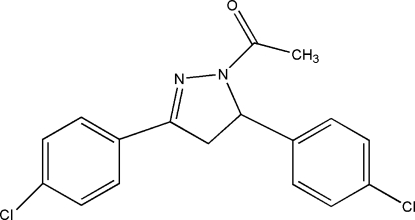


## Experimental

### 

#### Crystal data

C_17_H_14_Cl_2_N_2_O
*M* *_r_* = 333.20
Monoclinic, 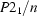

*a* = 6.0716 (9) Å
*b* = 13.160 (2) Å
*c* = 19.782 (3) Å
β = 98.412 (2)°
*V* = 1563.6 (4) Å^3^
*Z* = 4
Mo *K*α radiation
μ = 0.42 mm^−1^
*T* = 100 K
0.55 × 0.38 × 0.21 mm

#### Data collection

Bruker APEXII CCD diffractometer
Absorption correction: multi-scan (*APEX2*; Bruker, 2008 ▶) *T*_min_ = 0.803, *T*_max_ = 0.917
18906 measured reflections
4809 independent reflections
4141 reflections with *I* > 2σ(*I*)
*R*_int_ = 0.026

#### Refinement

*R*[*F* ^2^ > 2σ(*F* ^2^)] = 0.037
*wR*(*F* ^2^) = 0.111
*S* = 1.44
4809 reflections
200 parameters
H-atom parameters constrained
Δρ_max_ = 0.39 e Å^−3^
Δρ_min_ = −0.26 e Å^−3^

### 

Data collection: *APEX2* (Bruker, 2008 ▶); cell refinement: *SAINT* (Bruker, 2008 ▶); data reduction: *SAINT*; program(s) used to solve structure: *SHELXTL* (Sheldrick, 2008 ▶); program(s) used to refine structure: *SHELXTL*; molecular graphics: *SHELXTL*; software used to prepare material for publication: *SHELXTL*.

## Supplementary Material

Crystal structure: contains datablocks global, I. DOI: 10.1107/S1600536810025584/fj2320sup1.cif

Structure factors: contains datablocks I. DOI: 10.1107/S1600536810025584/fj2320Isup2.hkl

Additional supplementary materials:  crystallographic information; 3D view; checkCIF report

## Figures and Tables

**Table 1:** Hydrogen-bond geometry (Å, °)

*D*—H⋯*A*	*D*—H	H⋯*A*	*D*⋯*A*	*D*—H⋯*A*
C8—H8⋯Cl1^i^	0.93	2.80	3.5996 (13)	145
C9—H9⋯O1^ii^	0.93	2.59	3.4620 (15)	156

Symmetry codes: (i) 
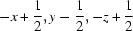
; (ii) 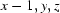.

**Fig. 1.**
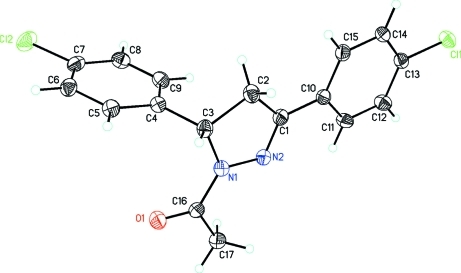
Molecular structure of (I), C17H14C12N2O, showing the atom labeling scheme and 50% probability displacement ellipsoids.

**Fig. 2.**
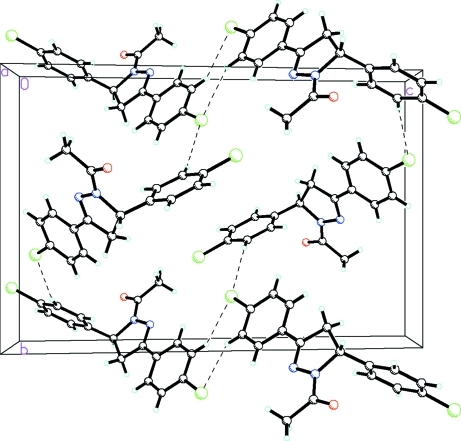
Packing diagram of the title compound, (I), viewed down the a axis.

**Fig. 3.**
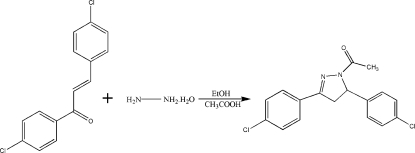
Reaction scheme of (I).

## supplementary crystallographic information

### Comment

Due to the interesting activity of variously substituted pyrazolines as biological agents considerable attention has been focused on this class of compounds. They are used as antitumor, antibacterial, antifungal, antiviral, antiparasitic, anti-tubercular and insecticidal agents (Hes *et al.*, 1978; Manna *et al.* 2005; Amir *et al.*, 2008).Some of these compounds have also anti-inflammatory, anti-diabetic, anaesthetic and analgesic properties Regaila *et al.*, 1979). Among the existing various pyrazoline type derivatives, 1-acetyl-pyrazolines have been identified as one of the most promising scaffolds. In the field of medicinal chemistry, 1-acetyl-pyrazoline derivatives were found to display anticancer and anti-inflammatory activities. In addition, pyrazolines have played a crucial part in the development of theory in heterocyclic chemistry and also used extensively in organic synthesis (Klimova *et al.*, 1999 & Bhaskarreddy *et al.*, 1997). In continuation of our work on pyrazoline derivatives (Samshuddin *et al.*, 2010, Fun *et al.*, 2010, Yathirajan *et al.*, 2007*a*,b, Butcher *et al.*, 2007) and in view of the importance of these derivatives, the title compound (I) is synthesized and its crystal structure is reported here.

In (I), two chloro-substituted benzene rings are bonded to opposite ends of an acetyl substituted pyrazole ring in a slightly distorted envelope conformation (Fig. 1). The dihedral angle between the mean planes of the benzene (C4–C9) and chloro substituted benzene rings (C10–C15) with the pyrazole ring are 75.97 ° and 16.63 ° respectively. Two weak C—H···O intermolecular hydrogen bonds (Table 1) and π-π stacking interactions (Table 2) are observed which contribute to crystal packing stability (Fig. 2).

### Experimental

A mixture of (2E)-1,3-bis(4-chlorophenyl)prop-2-en-1-one (2.77 g, 0.01 mol) and hydrazine hydrate (0.5 ml, 0.01 mol) in 25 ml e thanol in presence of 2 ml glacial acetic acid was refluxed for 5 h (Fig. 3). The reaction mixture was cooled and poured into 50 ml ice-cold water. The precipitate was collected by filtration and purified by recrystallization from ethanol. The single-crystal was grown from DMF by slow evaporation method and yield of the compound was 84%.(m.p. 376 K). Analytical data: Found (Calculated): C %: 61.21(61.28); H%: 4.25 (4.23); N%: 8.35(8.41).

### Refinement

All of the H atoms were placed in their calculated positions and then refined using the riding model with C—H = 0.93–0.98 Å, and with *U*_iso_(H) = 1.17–1.49*U*_eq_(C).

### Crystal data

**Table:**

C_17_H_14_Cl_2_N_2_O	*F*(000) = 688
*M**_r_* = 333.20	*D*_x_ = 1.415 Mg m^−^^3^
Monoclinic, *P*2_1_/*n*	Mo *K*α radiation, λ = 0.71073 Å
Hall symbol: -P 2yn	Cell parameters from 6706 reflections
*a* = 6.0716 (9) Å	θ = 2.6–30.8°
*b* = 13.160 (2) Å	µ = 0.42 mm^−^^1^
*c* = 19.782 (3) Å	*T* = 100 K
β = 98.412 (2)°	Block, colourless
*V* = 1563.6 (4) Å^3^	0.55 × 0.38 × 0.21 mm
*Z* = 4	

### Data collection

**Table:**

Bruker APEXII CCD diffractometer	4809 independent reflections
Radiation source: fine-focus sealed tube	4141 reflections with *I* > 2σ(*I*)
graphite	*R*_int_ = 0.026
ω scans	θ_max_ = 31.3°, θ_min_ = 1.9°
Absorption correction: multi-scan (*APEX2*; Bruker, 2008)	*h* = −8→8
*T*_min_ = 0.803, *T*_max_ = 0.917	*k* = −18→18
18906 measured reflections	*l* = −28→28

### Refinement

**Table:**

Refinement on *F*^2^	Primary atom site location: structure-invariant direct methods
Least-squares matrix: full	Secondary atom site location: difference Fourier map
*R*[*F*^2^ > 2σ(*F*^2^)] = 0.037	Hydrogen site location: inferred from neighbouring sites
*wR*(*F*^2^) = 0.111	H-atom parameters constrained
*S* = 1.44	*w* = 1/[σ^2^(*F*_o_^2^) + (0.0475*P*)^2^] where *P* = (*F*_o_^2^ + 2*F*_c_^2^)/3
4809 reflections	(Δ/σ)_max_ = 0.001
200 parameters	Δρ_max_ = 0.39 e Å^−^^3^
0 restraints	Δρ_min_ = −0.26 e Å^−^^3^

### Special details

**Table:**

Geometry. All e.s.d.'s (except the e.s.d. in the dihedral angle between two l.s. planes) are estimated using the full covariance matrix. The cell e.s.d.'s are taken into account individually in the estimation of e.s.d.'s in distances, angles and torsion angles; correlations between e.s.d.'s in cell parameters are only used when they are defined by crystal symmetry. An approximate (isotropic) treatment of cell e.s.d.'s is used for estimating e.s.d.'s involving l.s. planes.
Refinement. Refinement of *F*^2^ against ALL reflections. The weighted *R*-factor *wR* and goodness of fit *S* are based on *F*^2^, conventional *R*-factors *R* are based on *F*, with *F* set to zero for negative *F*^2^. The threshold expression of *F*^2^ > σ(*F*^2^) is used only for calculating *R*-factors(gt) *etc*. and is not relevant to the choice of reflections for refinement. *R*-factors based on *F*^2^ are statistically about twice as large as those based on *F*, and *R*- factors based on ALL data will be even larger.

### Fractional atomic coordinates and isotropic or equivalent isotropic displacement parameters (Å^2^)

**Table:**

	*x*	*y*	*z*	*U*_iso_*/*U*_eq_	
Cl1	−0.05600 (5)	0.67498 (2)	0.037299 (16)	0.02593 (9)	
Cl2	1.07200 (6)	0.31318 (3)	0.545917 (17)	0.03442 (10)	
O1	1.42292 (14)	0.36398 (8)	0.25597 (5)	0.0284 (2)	
N1	1.10312 (16)	0.44665 (8)	0.22241 (5)	0.0223 (2)	
N2	0.90756 (16)	0.45866 (8)	0.17716 (5)	0.0215 (2)	
C1	0.80954 (19)	0.53952 (9)	0.19412 (6)	0.0198 (2)	
C2	0.93583 (19)	0.59546 (9)	0.25404 (6)	0.0227 (2)	
H2A	0.8438	0.6066	0.2895	0.027*	
H2B	0.9900	0.6604	0.2401	0.027*	
C3	1.13032 (19)	0.52236 (9)	0.27851 (6)	0.0205 (2)	
H3	1.2730	0.5578	0.2802	0.025*	
C4	1.11491 (18)	0.47227 (8)	0.34639 (6)	0.0185 (2)	
C5	1.29877 (19)	0.46775 (9)	0.39706 (6)	0.0204 (2)	
H5	1.4322	0.4973	0.3895	0.024*	
C6	1.2861 (2)	0.41974 (9)	0.45879 (6)	0.0226 (2)	
H6	1.4100	0.4164	0.4924	0.027*	
C7	1.0866 (2)	0.37695 (9)	0.46945 (6)	0.0227 (2)	
C8	0.9003 (2)	0.38125 (9)	0.42023 (6)	0.0239 (2)	
H8	0.7665	0.3528	0.4285	0.029*	
C9	0.91492 (19)	0.42834 (9)	0.35855 (6)	0.0218 (2)	
H9	0.7908	0.4308	0.3249	0.026*	
C10	0.59722 (19)	0.57204 (8)	0.15606 (6)	0.0189 (2)	
C11	0.4946 (2)	0.51628 (9)	0.10008 (6)	0.0230 (2)	
H11	0.5614	0.4571	0.0874	0.028*	
C12	0.2948 (2)	0.54783 (9)	0.06325 (6)	0.0236 (2)	
H12	0.2282	0.5108	0.0257	0.028*	
C13	0.19554 (19)	0.63577 (9)	0.08334 (6)	0.0200 (2)	
C14	0.2917 (2)	0.69243 (9)	0.13857 (6)	0.0214 (2)	
H14	0.2227	0.7510	0.1515	0.026*	
C15	0.4939 (2)	0.66028 (9)	0.17462 (6)	0.0214 (2)	
H15	0.5610	0.6982	0.2116	0.026*	
C16	1.2506 (2)	0.37105 (9)	0.21546 (6)	0.0233 (2)	
C17	1.1866 (2)	0.29880 (10)	0.15692 (6)	0.0307 (3)	
H17A	1.3052	0.2512	0.1549	0.046*	
H17B	1.1597	0.3363	0.1149	0.046*	
H17C	1.0539	0.2628	0.1636	0.046*	

### Atomic displacement parameters (Å^2^)

**Table:**

	*U*^11^	*U*^22^	*U*^33^	*U*^12^	*U*^13^	*U*^23^
Cl1	0.02224 (16)	0.02398 (16)	0.02988 (17)	0.00321 (10)	−0.00184 (11)	−0.00217 (11)
Cl2	0.0471 (2)	0.03506 (19)	0.02341 (17)	−0.00037 (14)	0.01295 (14)	0.00586 (12)
O1	0.0234 (5)	0.0361 (5)	0.0260 (5)	0.0063 (4)	0.0045 (3)	0.0069 (4)
N1	0.0212 (5)	0.0270 (5)	0.0184 (5)	0.0046 (4)	0.0016 (4)	−0.0002 (4)
N2	0.0211 (5)	0.0251 (5)	0.0181 (5)	0.0027 (4)	0.0023 (4)	0.0016 (4)
C1	0.0212 (5)	0.0202 (5)	0.0181 (5)	−0.0008 (4)	0.0034 (4)	0.0016 (4)
C2	0.0245 (6)	0.0202 (6)	0.0229 (6)	0.0004 (4)	0.0010 (4)	0.0009 (4)
C3	0.0194 (5)	0.0212 (5)	0.0203 (5)	−0.0013 (4)	0.0011 (4)	0.0014 (4)
C4	0.0183 (5)	0.0175 (5)	0.0194 (5)	−0.0005 (4)	0.0020 (4)	0.0000 (4)
C5	0.0171 (5)	0.0214 (5)	0.0223 (5)	−0.0033 (4)	0.0015 (4)	−0.0019 (4)
C6	0.0246 (6)	0.0223 (5)	0.0201 (5)	−0.0008 (4)	0.0004 (4)	−0.0023 (4)
C7	0.0301 (6)	0.0200 (5)	0.0194 (5)	0.0003 (4)	0.0087 (5)	−0.0005 (4)
C8	0.0205 (5)	0.0227 (6)	0.0300 (6)	−0.0016 (4)	0.0085 (5)	0.0010 (5)
C9	0.0168 (5)	0.0218 (5)	0.0266 (6)	−0.0009 (4)	0.0021 (4)	−0.0002 (4)
C10	0.0200 (5)	0.0183 (5)	0.0188 (5)	−0.0001 (4)	0.0046 (4)	0.0024 (4)
C11	0.0228 (6)	0.0209 (5)	0.0255 (6)	0.0024 (4)	0.0044 (4)	−0.0043 (4)
C12	0.0227 (6)	0.0218 (6)	0.0259 (6)	0.0000 (4)	0.0018 (4)	−0.0053 (4)
C13	0.0188 (5)	0.0195 (5)	0.0219 (6)	0.0003 (4)	0.0037 (4)	0.0019 (4)
C14	0.0257 (6)	0.0173 (5)	0.0215 (6)	0.0034 (4)	0.0043 (4)	0.0011 (4)
C15	0.0273 (6)	0.0187 (5)	0.0177 (5)	0.0009 (4)	0.0018 (4)	0.0010 (4)
C16	0.0240 (6)	0.0272 (6)	0.0202 (6)	0.0055 (5)	0.0083 (4)	0.0064 (4)
C17	0.0373 (7)	0.0318 (7)	0.0239 (6)	0.0122 (6)	0.0069 (5)	0.0006 (5)

### Geometric parameters (Å, °)

**Table:**

Cl1—C13	1.7382 (12)	C6—H6	0.9300
Cl2—C7	1.7435 (12)	C7—C8	1.3817 (17)
O1—C16	1.2249 (15)	C8—C9	1.3829 (17)
N1—C16	1.3588 (15)	C8—H8	0.9300
N1—N2	1.3875 (14)	C9—H9	0.9300
N1—C3	1.4826 (15)	C10—C15	1.3946 (16)
N2—C1	1.2875 (14)	C10—C11	1.3968 (16)
C1—C10	1.4587 (16)	C11—C12	1.3842 (17)
C1—C2	1.5063 (16)	C11—H11	0.9300
C2—C3	1.5449 (16)	C12—C13	1.3895 (16)
C2—H2A	0.9700	C12—H12	0.9300
C2—H2B	0.9700	C13—C14	1.3799 (17)
C3—C4	1.5110 (16)	C14—C15	1.3923 (17)
C3—H3	0.9800	C14—H14	0.9300
C4—C5	1.3881 (16)	C15—H15	0.9300
C4—C9	1.3972 (16)	C16—C17	1.5049 (18)
C5—C6	1.3871 (16)	C17—H17A	0.9600
C5—H5	0.9300	C17—H17B	0.9600
C6—C7	1.3795 (17)	C17—H17C	0.9600
			
C16—N1—N2	122.18 (10)	C7—C8—H8	120.4
C16—N1—C3	124.39 (10)	C9—C8—H8	120.4
N2—N1—C3	113.42 (9)	C8—C9—C4	120.47 (11)
C1—N2—N1	108.10 (10)	C8—C9—H9	119.8
N2—C1—C10	121.01 (11)	C4—C9—H9	119.8
N2—C1—C2	114.05 (10)	C15—C10—C11	118.71 (11)
C10—C1—C2	124.93 (10)	C15—C10—C1	120.40 (11)
C1—C2—C3	102.71 (9)	C11—C10—C1	120.88 (10)
C1—C2—H2A	111.2	C12—C11—C10	120.98 (11)
C3—C2—H2A	111.2	C12—C11—H11	119.5
C1—C2—H2B	111.2	C10—C11—H11	119.5
C3—C2—H2B	111.2	C11—C12—C13	118.86 (11)
H2A—C2—H2B	109.1	C11—C12—H12	120.6
N1—C3—C4	110.94 (9)	C13—C12—H12	120.6
N1—C3—C2	100.81 (9)	C14—C13—C12	121.71 (11)
C4—C3—C2	113.97 (9)	C14—C13—Cl1	119.46 (9)
N1—C3—H3	110.3	C12—C13—Cl1	118.82 (9)
C4—C3—H3	110.3	C13—C14—C15	118.71 (11)
C2—C3—H3	110.3	C13—C14—H14	120.6
C5—C4—C9	119.03 (10)	C15—C14—H14	120.6
C5—C4—C3	120.83 (10)	C14—C15—C10	121.01 (11)
C9—C4—C3	120.13 (10)	C14—C15—H15	119.5
C6—C5—C4	120.86 (11)	C10—C15—H15	119.5
C6—C5—H5	119.6	O1—C16—N1	120.13 (12)
C4—C5—H5	119.6	O1—C16—C17	123.72 (11)
C7—C6—C5	118.91 (11)	N1—C16—C17	116.14 (11)
C7—C6—H6	120.5	C16—C17—H17A	109.5
C5—C6—H6	120.5	C16—C17—H17B	109.5
C6—C7—C8	121.49 (11)	H17A—C17—H17B	109.5
C6—C7—Cl2	119.10 (10)	C16—C17—H17C	109.5
C8—C7—Cl2	119.39 (9)	H17A—C17—H17C	109.5
C7—C8—C9	119.23 (11)	H17B—C17—H17C	109.5
			
C16—N1—N2—C1	−176.08 (10)	Cl2—C7—C8—C9	−177.42 (9)
C3—N1—N2—C1	5.01 (13)	C7—C8—C9—C4	−0.83 (18)
N1—N2—C1—C10	−179.54 (10)	C5—C4—C9—C8	0.14 (17)
N1—N2—C1—C2	1.70 (13)	C3—C4—C9—C8	179.19 (11)
N2—C1—C2—C3	−7.14 (13)	N2—C1—C10—C15	−179.43 (11)
C10—C1—C2—C3	174.16 (10)	C2—C1—C10—C15	−0.81 (17)
C16—N1—C3—C4	−66.76 (14)	N2—C1—C10—C11	−0.06 (17)
N2—N1—C3—C4	112.11 (10)	C2—C1—C10—C11	178.56 (11)
C16—N1—C3—C2	172.18 (11)	C15—C10—C11—C12	0.38 (18)
N2—N1—C3—C2	−8.95 (12)	C1—C10—C11—C12	−179.00 (11)
C1—C2—C3—N1	8.80 (11)	C10—C11—C12—C13	−0.77 (18)
C1—C2—C3—C4	−110.09 (11)	C11—C12—C13—C14	0.43 (18)
N1—C3—C4—C5	113.38 (12)	C11—C12—C13—Cl1	−179.69 (9)
C2—C3—C4—C5	−133.67 (11)	C12—C13—C14—C15	0.28 (18)
N1—C3—C4—C9	−65.65 (13)	Cl1—C13—C14—C15	−179.60 (9)
C2—C3—C4—C9	47.30 (15)	C13—C14—C15—C10	−0.67 (18)
C9—C4—C5—C6	0.53 (17)	C11—C10—C15—C14	0.35 (17)
C3—C4—C5—C6	−178.51 (11)	C1—C10—C15—C14	179.73 (11)
C4—C5—C6—C7	−0.50 (17)	N2—N1—C16—O1	178.94 (10)
C5—C6—C7—C8	−0.21 (18)	C3—N1—C16—O1	−2.28 (18)
C5—C6—C7—Cl2	178.08 (9)	N2—N1—C16—C17	−1.78 (16)
C6—C7—C8—C9	0.87 (18)	C3—N1—C16—C17	177.00 (11)

### Hydrogen-bond geometry (Å, °)

**Table:**

*D*—H···*A*	*D*—H	H···*A*	*D*···*A*	*D*—H···*A*
C8—H8···Cl1^i^	0.93	2.80	3.5996 (13)	145
C9—H9···O1^ii^	0.93	2.59	3.4620 (15)	156

Symmetry codes: (i) −*x*+1/2, *y*−1/2, −*z*+1/2; (ii) *x*−1, *y*, *z*.

### Table 2 Cg···Cg π stacking interactions,

Cg1 is the centroid of the ring N1/N2/C1/C2/C3, Cg2 is the centroid of the ring C4–C9 and Cg3 is the centroid of the ring C10–C125.

**Table:**

	*Cg*X···*Cg*Y (Å)	Cg1···Perp (Å)	Cg2···Perp (Å)	Cg3···Perp (Å)
Cg1···Cg2^i^	3.774 (4)	-0.283 (8)	-2.559 (6)	
Cg1···Cg3^ii^	3.716 (7)	-3.572 (2)		-3.594 (1)

Symmetry codes: (i) x, y, z; (ii) 1 + x, y, z.

## References

[bb1] Amir, M., Kumar, H. & Khan, S. A. (2008). *Bioorg. Med. Chem. Lett.* **18**, 918–922. 10.1016/j.bmcl.2007.12.043 18182288

[bb2] Anuradha, N., Thiruvalluvar, A., Mahalinga, M. & Butcher, R. J. (2008). *Acta Cryst.* E**64**, o2160. 10.1107/S1600536808033837 PMC2959565 21581020

[bb3] Bhaskarreddy, D., Chandrasekhar, B. N., Padmavathi, V. & Sumathi, R. P. (1997). *Synthesis*, **3**, 491–494.

[bb4] Bruker (2008). *APEX2* and *SAINT* Bruker AXS Inc., Madison, Wisconsin, USA

[bb5] Butcher, R. J., Jasinski, J. P., Prasad, D. J., Narayana, B. & Yathirajan, H. S. (2007). *Acta Cryst.* E**63**, o4005–o4006.

[bb6] Fun, H.-K., Hemamalini, M., Samshuddin, S., Narayana, B. & Yathirajan, H. S. (2010). *Acta Cryst.* E**66**, o582–o583. 10.1107/S1600536810004435 PMC2983722 21580348

[bb7] Hes, R. V., Wellinga, K. & Grosscurt, A. C. (1978). *J. Agric. Food Chem.* **26**, 915–918.

[bb8] Jian, F.-F. & Wang, J. (2006). *Acta Cryst.* E**62**, o5303–o5304.

[bb9] Jian, F.-F., Wang, J. & Xiao, H.-L. (2006). *Acta Cryst.* E**62**, o4771–o4772.

[bb10] Klimova, E. I., Marcos, M., Klimova, T. B., Cecilio, A. T., Ruben, A. T. & Lena, R. R. (1999). *J. Organomet. Chem.* **585**, 106–111.

[bb11] Lu, Z.-K., Diao, H.-L., Li, S. & He, B. (2008). *Acta Cryst.* E**64**, o1638. 10.1107/S160053680801979X PMC2962117 21203327

[bb12] Manna, F., Chimenti, F., Fioravanti, R., Bolasco, A., Secci, D., Chimenti, P., Ferlini, C. & Scambia, G. (2005). *Bioorg. Med. Chem. Lett.* **15**, 4632–4635. 10.1016/j.bmcl.2005.05.067 16099651

[bb13] Regaila, H. A., El-Bayonk, A. K. & Hammad, M. (1979). *Egypt. J. Chem.* **20**, 197–202.

[bb14] Samshuddin, S., Narayana, B., Yathirajan, H. S., Safwan, A. P. & Tiekink, E. R. T. (2010). *Acta Cryst.* E**66**, o1279–o1280. 10.1107/S1600536810015795 PMC2979444 21579379

[bb15] Sheldrick, G. M. (2008). *Acta Cryst.* A**64**, 112–122. 10.1107/S0108767307043930 18156677

[bb16] Wang, S.-F., Zhu, W., Yang, X. & Zhou, L.-J. (2005). *Acta Cryst.* E**61**, o3985–o3986.

[bb17] Yathirajan, H. S., Bindya, S., Sarojini, B. K., Narayana, B. & Bolte, M. (2007*a*). *Acta Cryst.* E**63**, o2718.

[bb18] Yathirajan, H. S., Bindya, S., Sarojini, B. K., Narayana, B. & Bolte, M. (2007*b*). *Acta Cryst.* E**63**, o2566.

